# Scouting as a Strategy in Support of Mental Health Development Through the Formation of Sense of Self-Efficacy

**DOI:** 10.3390/brainsci14121268

**Published:** 2024-12-17

**Authors:** Roman Ryszard Szałachowski, Weronika Własak, Wioletta Tuszyńska-Bogucka

**Affiliations:** 1 Faculty of Social Sciences, University of Szczecin, 71-017 Szczecin, Poland; 2 Faculty of Human Sciences, University of Economics and Innovation, 20-209 Lublin, Poland

**Keywords:** personal competence, social development, a sense of self-efficacy, stress coping styles, scouting, non-formal education

## Abstract

Background: This research project examining the moderating role of the Scout Movement in supporting mental health through the shaping of personal competence is based on Bandura’s conception of social development (social cognitive theory) in terms of generating a sense of general self-efficacy. Methods: This research examined the moderating value of Scouting with regard to the connection between self-esteem and a sense of efficacy and styles of coping with stress in a group of 683 volunteers. Results: The results suggest that Scouting is a moderator of the relationship between the intensity of an emotion-focused stress coping style and a sense of self-efficacy—being a Scout intensifies the blocking effect of self-esteem on emotions in stressful situations, which can positively influence emotion regulation. Conclusions: The features described suggest the need to research Scouting as a non-formal education strategy to support the development of young people’s mental health in different theoretical and methodological contexts. This work provides conclusions regarding understanding the role of Scouting as a moderator in coping with stress and, consequently, ensuring good mental health. It detailed the knowledge pertaining to specific mechanisms thanks to which Scouting can influence the development of emotional regulation and adaptive response to stressful situations.

## 1. Introduction

One of the factors that is relevant to youth development and functioning is membership in the Scout Movement. International Scouting is inherently linked to the idea of non-formal education, characterized by innovative, diverse, and contextualized learning strategies for children, adolescents, and adults worldwide in settings that are as close to natural as possible [1,2,3,4]. Scouting is based on education, teaching such values as respect, responsibility, loyalty, an attitude of service and respect for the environment, and learning through work, always alongside other scouts and adult supervisors. Scouting programs focus on the participants’ centers of interest involving a direct contact with nature aimed at developing mental, social, physical, affective, and spiritual spheres [1]. The non-formal nature of Scouting locates it in the individual’s free time—according to the classification by Quintano [1], it is placed in the category of utilitarian time, free from formal obligations, but based on voluntary commitments. Oropesa [1], in an analysis of leisure time and its impact on the evolutionary development of the adolescent, presents leisure time as the ideal context to satisfy the needs for autonomy, competence, and relationships with other people, essential for the full development of the adolescent as a person. Scouting fosters qualities such as responsibility, self-discipline, and self-control. These attributes are cultivated through group activities perceived as adventurous and distinct from routine schooling, promoting their internalization. This allows teenagers to cope more independently, to take more responsibility for their actions, and to make more informed choices, which in turn may enhance their self-confidence and self-assurance [5].

**Figure 1:**
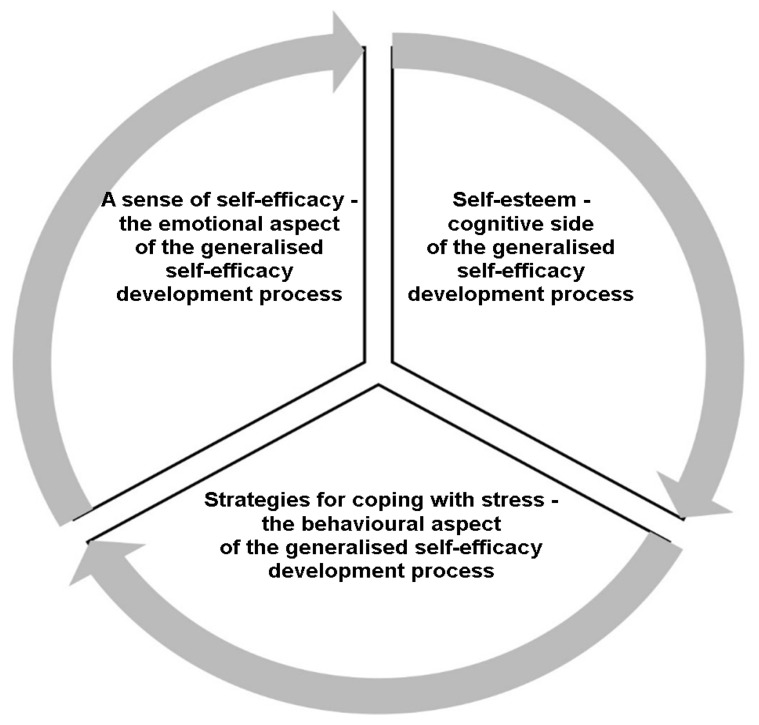
Bandura’s operationalization of personal development as a sense of general self-efficacy.

The characteristics described suggest the need to research the Scout Movement as a non-formal education strategy for the personal, social, and academic development of adolescents in different theoretical and methodological contexts. Given the increasing focus on adolescent mental health, understanding how Scouting contributes to emotional regulation and resilience is crucial. Our research aims to provide new knowledge on the relationship between the different variables examined in terms of moderation. This research project was based on Bandura’s [6] concept of social development (social cognitive theory) as the creation of a sense of general self-efficacy. The choice was dictated by a good adaptation of the theory to the specific nature of work in Scouting—the method is based on the implementation of various tasks in small peer groups and the use of a stimulating learning-by-action program. For the purposes of this research project, it has been operationalized as a set of characteristics/abilities, as follows:

(1) a sense of self-efficacy, which refers to the individual’s belief in their ability to exhibit behaviors necessary to achieve specific outcomes [6,7], so it reflects confidence in the individual’s ability to exert control over their motivation, behavior, and social environment, giving it the character of the emotional aspect of a sense of general self-efficacy—the ability to manage emotions internally rather than externally. A person who is confident in their self-efficacy is more motivated to overcome any encountered obstacles, has a more positive attitude towards tasks, and is sure that they can handle them;
(2) the ability to use an effective stress coping style (located mainly in the approach), task-oriented behavior that in turn refers to the behavioral (skills) aspect, defined by Bandura as coping with tasks in an energetic way, without stress and disappointment;
(3) self-esteem, considered as the cognitive aspect of a sense of general self-efficacy (Figure 1).

It seems that this operationalization is consistent with Bandura’s postulate to explain the perception of self-regulation as a socio-cognitive process [6,8]. 

Self-esteem can have a significant impact on an individual’s behavior. Numerous studies have shown that the way a person sees themselves can translate into reactions in a stressful situation [9]. Self-esteem is an important mechanism in the secondary cognitive interpretation of resources. This is especially important in the speculation phase regarding estimating the chances of reducing the level of stress experienced, which relates to actual coping with stress. High self-esteem acts as a buffer protecting a person against the negative effects of stressful situations. Thanks to high self-esteem, an individual can adequately determine their skills and preference task-focused styles of coping with stress [9]. 

Coping mechanisms can also be analyzed in terms of the style of reaction to stress caused by various problems. From the age of sixteen to twenty-one, these struggles may be related to plans, a crisis of values, further education and choice of profession, spending free time, finding a job, or the style of functioning in interpersonal relationships [10]. These issues are more likely to occur in individuals whose environmental and sociocultural “constraints” make the resolve impossible or difficult for them. People can only use three strategies. The first one is an active tactic, which is shaped by the search for information and advice to obtain the information needed to resolve the dilemma. The second one is the internal-reflective strategy, which involves analyzing one’s own reactions, possibilities, and life situations. The third one, defined as passive, is characterized by the individual’s withdrawal, built out of their defense mechanisms, a regression and retreat into oneself. It is believed that the first two strategies are most often used by people with high self-esteem and a veracious reception of the social world, while the third one is used by people with low self-esteem. A passive strategy may lead to developmentally negative phenomena, maladaptive behaviors, or depression [11,12]. 

Testing his own theory, the author concludes that psychological procedures cause changes in behaviors by altering the level and strength of self-efficacy. Self-efficacy was an equally accurate predictor of performance on tasks of varying levels of difficulty with different threats, and regardless of whether changes in self-efficacy were induced by active mastery (enactive mastery) or only by vicarious experience (vicarious experience alone) [13].

Based on the literature, three research hypotheses were formulated regarding differences in severity: (H1) sense of self-efficacy; (H2) self-esteem; (H3) tendency to choose a stress coping style (3.1. task-focused, 3.2. emotion-focused, and 3.3. avoidance-focused) between individuals, members and non-members of the Scout Movement. Additionally, interaction hypotheses were formulated regarding differences in the type of relationship between: (H4) coping with stress (4.1. task-focused, 4.2. emotion-focused, and 4.3. avoidance-focused) and self-efficacy moderated by belonging to the Scout Movement, (H5) coping with stress (5.1. task-focused, 5.2. emotion-focused, and 5.3. avoidance-focused) and self-esteem moderated by belonging to the Scout Movement, (H6) self-esteem and a sense of self-efficacy moderated by belonging to the Scout Movement. 

## 2. Materials and Methods

### 2.1. Measurement of Variables

The selection of variables was determined by their constant presence in studies across the world and their good positioning in the proposed theoretical model. They optimize the measurement of designated variables and ensure the reliability of the obtained results thanks to good psychometric properties. Polish adaptations of the tools used consider cultural or contextual factors, and the behaviors or preferences included in the original questions, and in the adaptation, were balanced in terms of the degree of positivity–negativity spectrum and the level of burden of social approval [14,15,16,17,18]. The studied features are also relatively resistant to cultural differences [19,20], which confirms their wide use in research in various countries and cultures [21,22,23,24,25,26,27] All in all, it can be said that the use of verified tools (GSES, SES, and CISS) ensures the reliability of the measurement of selected variables.

The Generalised Self-Efficacy Scale (GSES) by Schwarzer, Jerusalem and Juczyński is used to measure the strength of an individual’s general belief in the effectiveness of coping with difficult situations and obstacles—the sense of self-efficacy [14,15]. The questionnaire includes ten questions. The respondent is asked to respond to them using a four-point scale ranging from 1—no to 4—yes. It is assumed that higher scores on the scale indicate a higher overall belief of the respondent in the effectiveness of coping with difficult situations and obstacles. The α–Cronbach’s reliability coefficient stood at 0.88. 

Rosenberg’s Self-Esteem Scale (SES) is used to determine the overall level of self-esteem. The scale includes ten questions [16]. The respondent is asked to indicate to what extent they agree with a statement on a four-point scale from 1—strongly agree to 4—strongly disagree. The higher the obtained score, the higher the level of the individual’s overall self-esteem. The α–Cronbach’s reliability coefficient stood at 0.9.

The Coping in Stressful Situations (CISS) questionnaire by Endler and Parker [17] is designed to diagnose stress coping styles [18]. It includes 48 statements addressing the individual’s various courses of action in stressful situations. The respondent answers them using a five-point scale ranging from 1—never to 5—very often. The results are represented on three scales, signaling a tendency to choose a particular style of coping with stress as follows: task-focused style; emotion-focused style; and avoidance-focused style. The highest score from the scales shows how an individual is going to cope with stress (which style they choose most often). The α–Cronbach’s reliability coefficient stood at 0.89. 

### 2.2. Participants

A total of 683 people participated in the research, mainly women (*n*_w_ = 485; 71%) and to a lesser extent men (*n*_m_ = 198; 29%). The participants were members of the Polish Scouting and Guiding Association [The Polish Scouting and Guiding Association (ZHP) is a member of the World Organization of the Scout Movement (WOSM), the World Association of Girl Guides and Girl Scouts (WAGGGS) and the International Scout and Guide Fellowship (ISGF)], hereafter called the Scout Movement or Scouting (*n*_1_ = 324; 47%) and non-members (*n*_2_ = 359; 53%), as determined by declarations. The age of the research participants ranged from 16 to 21 years (*M* = 17.6; *SD* = 1.62). The largest number of participants lived in a small city of up to 50,000 inhabitants (30%), while the smallest number lived in a large city of 250,000 and more inhabitants (13%). Such a profile of the study group may evidence a greater readiness of women for considering this scope of problems at all (expressed by a willingness to participate in this study).

The age group choice was dictated by the characteristics of the group called ‘Wanderers’—the oldest Scout Movement group, undertaking tasks worthy of achievement and performing conscious service for the local environment. The most important elements of work with this methodologically distinguished group are achievement, wander, and service. In the Polish Scouting Association, the service element is present in every age group but, among ‘Wanderers’, it is a conscious and advanced quality. Members can help Polish Scouting Association through their activities but, thanks to their knowledge and experience, they are also active in the local community. It also involves being a conscious citizen. A deed for ‘Wanderers’ is a challenge that can be interpreted individually because it refers to various areas of human existence. This consolidates working on yourself with getting to know yourself, i.e., it ensures all-round development [28]. People of this age already have knowledge about many issues, have experienced difficult situations, and are looking for their identity. They develop a conscious self-image, thanks to which they better understand what is happening in their ‘wandering‘ team. All elements allow for spiritual, intellectual, emotional, and physical growth that can translate into self-esteem, self-efficacy, and coping with stress. Stress is common in the ‘wandering’ age because it is a time of change pertaining to the notion of travel. Individuals often must make many important decisions while participating in ‘Wanderers’ teams, which is associated with feeling numerous undesirable emotions. The evident importance of regulatory mechanisms resulted in the selection of a specific group.

### 2.3. Procedure

The research was conducted through an online questionnaire aimed at young people aged between 16 and 21 years (*M* = 17.61). The questionnaire was made available to social media users between June and December 2022. Before completing the battery of questions, participants were informed about the voluntary, anonymous, and scientific nature of the research and the security of data storage. They did not receive any remuneration for participating in the research. Respondents received information about the objectives of this study. They were also informed about the possibility of withdrawing from the research without giving a reason at any time and without suffering any consequences. They confirmed their informed consent to participate in this study by selecting “yes”. They were also informed that the obtained data is anonymized immediately after entering it into the data system (e.g., by removing the e-mail address or time stamp). A consent form was also included for parents of teenagers under 18 years of age, who had to consent to their participation in the research. The survey was verified by asking about possession of the Scout Cross, which proves membership in a Scout organization for at least three months.

### 2.4. Statistical Analysis

In the course of the work, statistical analyses of the differences between the research groups and a correlation analysis of the whole group’s performance were carried out. The final stage of the work was a bootstrapping analysis of moderation with a random selection of 5000 samples and the performance of simple slopes analysis (SSA). All analyses were performed in IBM SPSS Statistics 26 with the PROCESS 4.1 add-on. A 95% confidence interval (*p* < 0.05) was used to interpret the results for empirical verification of the formulated research hypotheses [29]. 

### 2.5. Descriptive Statistics of Variables and Preliminary Analyses

The Kolmogorov–Smirnov test was used to determine the distribution of the variables (Table 1).

The vast majority of the distributions of the examined variables were statistically significantly different from the normal distribution. The exceptions were as follows: the distribution of the variable emotion-based stress coping style for the whole group (*Z*(683) = 0.03; *p* > 0.05), the distribution of the variable avoidance-based stress coping style for those belonging to the Scout Movement (*Z*(324) = 0.04; *p* > 0.05), and the distribution of the variable emotion-based stress coping style for the non-member group (*Z*(359) = 0.05; *p* = 0.05), close to the normal distribution. In all other cases, the obtained kurtosis and skewness results were within the range < −1; 1 > resulting in the assumption that the deviation from the Gaussian distribution was not significant. In consequence, it was assumed that the nature of the distributions of all psychological variables was close to the normal distribution. 

Before proceeding to the actual calculations, all psychological variables were analyzed, checking individual assumptions related to the selection of appropriate tests. The elimination of extreme observations was performed based on a box plot analysis of the results of all collected questionnaires. In the next step of the preliminary analysis, the identification and elimination of influential outliers for the multiple regression models were carried out using Cook’s and Mahalanbois distance measures and impact values [30,31,32]. Failure to meet two of the three distance measures was used as a criterion for rejecting a given observation. The significance of differences in terms of the size of the groups compared was determined using a chi-squared test, which showed that there were no significant differences in terms of organizational membership (χ^2^ = 1.79; *p* > 0.05). In order to verify the assumption of normality of the distribution of the psychological variables, an analysis was conducted using the Shapiro–Wilk test, which supported this assumption. This allowed further analysis to be based on parametric statistical methods.

## 3. Results

### 3.1. Significance of Differences: Verification of Hypotheses 1–3

In order to compare the intensity of the examined variables in the compared groups, an analysis of the Student’s *t*-test for independent samples was performed. The analysis (Table 2) showed that members of the Scout Movement compared to non-members were characterized by significantly higher levels of self-efficacy (*t*(683) = 2.21; *p* < 0.05; *F* = 2.43), a significantly higher score in task-focused stress coping style (*t*(683) = 3.09; *p* < 0.01; *F* = 0.15), and a significantly lower score in avoidance-focused stress coping style (*t*(683) = 2.10; *p* < 0.05; *F* = 0.02). 

Among hypotheses regarding differences in severity: (H1) sense of self-efficacy, (H2) self-esteem, (H3) tendency to choose a stress coping style (3.1. task-based, 3.2. emotional, and 3.3. avoidant) between individuals, members and non-members of the Scout Movement, hypothesis H1, H3.1, and H3.3. were positively verified. This means that scouts have a higher sense of self-efficacy and a tendency to choose a task-focused coping style, and a lower tendency to choose an avoidance-focused style than non-scouts.

### 3.2. Correlation Analysis: Verification of Hypotheses 4–5

An analysis of the *r*-Pearson correlation coefficients (Table 3) was used to verify the hypotheses concerning the predicted relationships between self-efficacy, self-esteem, and stress coping styles scores (Hypotheses 4–5).

A coefficient value analysis indicated that a sense of self-efficacy correlated positively with self-esteem (*r* = 0.51; *p* < 0.001), with a task-focused stress coping style (*r* = 0.60; *p* < 0.001), and with an avoidance-focused coping style (*r* = 0.10; *p* < 0.05), and negatively with an emotion-focused stress coping style (*r* = −0.38; *p* < 0.001). In turn, self-esteem correlated positively with a task-focused stress coping style (*r* = 0.41; *p* < 0.001), and negatively with an emotion-focused stress coping style (*r* = −0.59; *p* < 0.001). All relationships were weak or moderate correlations. The value of the coefficients obtained justifies taking further steps to explain the relationships linking the examined variables, in this case a moderation analysis.

### 3.3. Analysis of Moderation: Verification of Hypotheses 4–6

**Figure 2:**
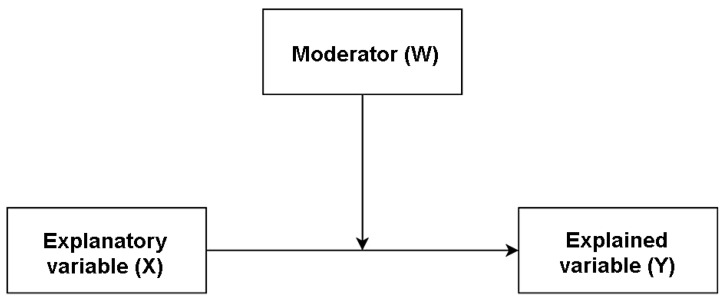
Diagram of the moderation analysis model.

In further calculations, moderation analysis (Figure 2) was carried out for seven models in order to provide empirical verification regarding the nature of the relationships between the study variables in two groups—scouts and non-scouts. A maximum likelihood estimation analysis method with a bootstrapping procedure was used to develop the hypothetical model. Bootstrapping is a method for obtaining robust estimates of standard errors and confidence intervals for regression coefficients. The bootstrap method is most useful as an alternative to parametric estimates by providing confidence intervals and predictions that are more robust to the nature of the data. Re-sampling is also a natural choice considering the observed deviations of the distributions of the analyzed variables from the normal distribution (Table 1). Also the type of hypothesis, the verification of which can be reduced to testing the estimation error using statistics that meet the bootstrap conditions, justifies this decision (https://www.ibm.com/docs/pl/spss-statistics/saas?topic=bootstrapping, accessed 10 September 2024) Taking a random sample can be treated as replacing a continuous random variable with an unknown distribution with a discrete variable with a known distribution—the bootstrap distribution (equivalent to the empirical distribution). Transformations of discrete variables are simpler than transformations of continuous variables because the distributions of statistics of discrete variables can be calculated automatically [33]. 

The moderation model assumes that X influences Y more or less strongly depending on the moderator W. The significance of moderation is based on the significance or otherwise of the coefficient associated with the interaction variable. In order to evaluate the model under test, different fit indices were considered as follows: the change in the coefficient R2, the non-standardized regression value between the variables for the member and non-member groups of the Scout Movement, and the value of the interaction effect.

Models 1 to 3 present the explanatory variable X, a sense of self-efficacy, and the explained variable Y, defined as a task-, avoidance-, or emotion-focused stress coping style influenced by a moderator, in this case belonging to the Scout Movement. Models 4 to 6 depict self-esteem, as explained by variable X, while explaining a task-focused, avoidance-focused, or emotion-focused stress coping style, variables influenced as a moderator by membership in the Scout Movement. Model 7 depicts self-esteem as the explained variable X and the explanatory variable Y, namely a sense of self-efficacy, this relationship is influenced as a moderator by belonging to the Scout Movement.

In order to empirically verify Hypotheses 4–5, a moderation analysis was performed using a bootstrapping method with a random selection of 5000 samples (Table 4).

The analysis of models 1–2 and 4–7 showed that all models were well-fitted (*F*-test value). However, the effects of the moderation analysis indicate the lack of significant differences between the interactive relationships between self-esteem and individual styles of coping with stress, self-efficacy, and the avoidant style and task-focused style of coping with stress, as well as between self-esteem and self-efficacy in groups of scouts and non-scouts. This means that membership in the Scout Movement is not a moderator and did not have a significant impact on the nature of these relationships.

Only in one case was a significant moderating effect shown. In Model 3, the results indicated an adequate fit of the model to the data (*F*(3.679) = 41.21; *p* < 0.001), which explained 15% of the variance in the dependent variable (*R*^2^ = 0.15). For both members of the Scout Movement (*B*_1_ = −1.21) and non-members (*B*_2_ = 0.77), the resulting non-standardized regression values between the explanatory and explained variable were statistically significant (*p* < 0.001). Membership in the Scout Movement significantly moderates the relationship between self-efficacy and emotion-focused stress coping (*Int* = 0.44; *p* < 0.05, 95% CI [0.09, 0.80]). 

Among interaction hypotheses regarding differences in the type of relationship between the following: (H4) stress coping styles (4.1. task-focused, 4.2. emotion-focused, and 4.3. avoidance-focused) and self-efficacy moderated by belonging to the Scout Movement, (H5) stress coping styles (4.1. task-focused, 4.2. emotion-focused, and 4.3. avoidance-focused) and self-esteem moderated by belonging (or not) to the Scout Movement, and (H6) self-esteem and a sense of self-efficacy moderated by belonging to the Scout Movement. Only Hypothesis 4.3 was positively verified. The effects of hypothesis verification are shown in the summary table (Table 5). 

**Figure 3:**
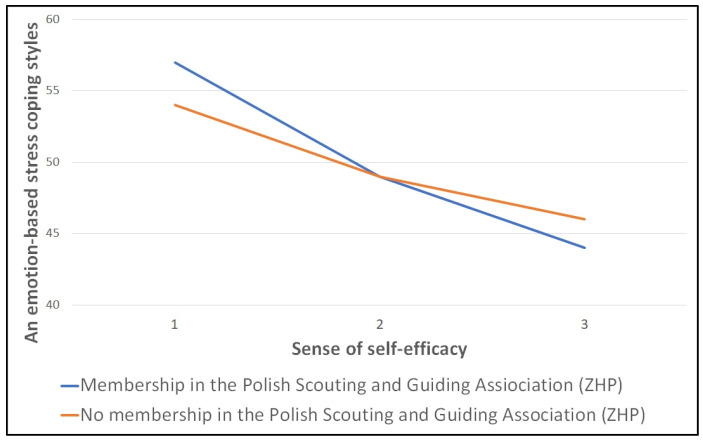
Simple slopes plot of the moderating role of Scout Movement membership in the relationship between a sense of self-efficacy and an emotion-based stress coping style (moderation plot for multilevel model).

Usually, the SSA approach is recommended for interpreting moderating effects [34,35]. SSA is a procedure that complements regression modelling to help examine and interpret ‘meaningful’ interactions. It involves plotting the relationship between the independent and dependent variables for certain discrete values of the moderating variable. To interpret the moderating effect, simple slopes were examined (Figure 3).

They indicate the results of two regressions—membership/no membership in the Scout Movement as a predictor of manner in coping with stress through emotions in relation to the intensity of a sense of self-efficacy. The analyses performed suggest that membership in the Scout Movement has a significant moderating effect on the relationship between variables X and Y, and therefore that, depending on membership/no membership, the relationship between a sense of self-efficacy and the intensity of the tendency to choose an emotion-based stress coping style varies (this is how the interaction works). In the remaining cases (Models 1–2, 4–6), there was no significant moderating effect, as the value of the interaction effects was not statistically significant.

## 4. Discussion

An analysis of the collected material allowed the verification of hypotheses and theoretical and practical conclusions regarding the important role of the Scout Movement in supporting the development of adolescents’ mental health through the formation of general self-efficacy as follows:

(1) The Scout Movement and a sense of self-efficacy. The results of the analysis confirmed Hypothesis 1—there is a difference in the level of a sense of self-efficacy between members and non-members of the Scout Movement. The mean level of a sense of self-efficacy among members of the Scout Movement (*M* = 29.81; *SD* = 4.68) is statistically significantly higher than among non-members (*M* = 28.99; *SD* = 4.98).
(2) The Scout Movement and the struggle with stress. The results of the analysis confirmed Hypothesis 3.2—there is a difference in the task-focused stress coping style between members and non-members of the Scout Movement. The mean of the task-focused stress coping style among scouts (*M* = 56.58; *SD* = 9.31) is statistically significantly higher than among non-scouts (*M* = 54.36; *SD* = 9.46). The results of the analysis also confirmed Hypothesis 3.3—there is a difference in the avoidance-focused stress coping style between members and non-members of the Scout Movement. The mean of the avoidance-focused stress coping style among scouts (*M* = 46.99; *SD* = 10.65) is statistically significantly lower than among non-scouts (*M* = 48.72; *SD* = 10.81). The question is as follows: for example, whether Scouting influences stress coping styles, or simply groups individuals who have a particular preference here? For this purpose, an analysis of the moderating effect was performed.
(3) The Scout Movement as a moderator. The results of the analysis showed that only Hypothesis 4.3 was confirmed, meaning that being part of the Scout Movement has a moderating status on the links between a sense of self-efficacy and an emotion-based stress coping style. The interaction effect value (*Int* = 0.44; *p* < 0.05) indicates the presence of significant moderation (95%CI [0.09; 0.80]) in the examined area. This means that the relationship between the studied variables is stronger among scouts compared to non-scouts.
(4) Only the emotional strategy for coping with stress was included in the interaction loop—it was a self-efficacy relationship. This means that in the remaining cases, neither self-awareness nor self-efficacy were associated with an increase in the tendency to choose a specific (avoidant or task-focused) strategy.
(5) The observed moderating relationship was relatively low. It can be explained by the fact that coping behaviors are not stable and therefore “state dependent” [36]; furthermore, stress levels are constantly altered [37]. There are individual differences in interpreting situations. According to the Lazarus stress model upon which stress measurement is based, predicting co-relations between behaviors is difficult.

The construction of the rules that form the basis of non-formal education, and the Scout Movement in particular, aims to have a positive impact on the personal and social skills of children and adolescents. Youth organizations that can offer young people long-term, extra-curricular leisure programs with aims that are educational in the broadest sense and are guided by adult supervisors have all the prerequisites for creating positive changes in the development of young people. The literature offers a substantial overview of research in this area, which indicates that adolescents who participate in the activities of youth organizations are more optimistic and self-confident, have more confidence in their ability to accomplish their goals, and hope to find a fulfilling job. These adolescents also have a higher sense of self-esteem, underpinned by a realistic insight. They are also more connected to their community, which signifies their healthy socialization development [4,5,38,39]. The impact of Scouting was associated with long-lasting positive emotions that provided energy and motivation to contribute to other important areas of life. Respondents stated that challenging themselves, performing tasks independently, and taking responsibility as part of the Scout Movement gave them the confidence and courage to deal with life and take responsibility in different situations. In the context of social development, it is worth mentioning the increase in confidence as a communicator, something the young people repeatedly mentioned in the interviews. The more introverted adolescents acquired self-confidence through positive communication experiences and became more open and courageous [5]. Here, it is appropriate to refer to extremely important studies directly relating to participation in the Scout Movement to mental health and psychological well-being. Dibben, Playford, and Mitchell [40] examined the long-term impact of being a scout on people’s mental health in the example of people born in England in 1958, concluding that participation in Scouting was associated with better mental health and fewer mental health inequalities at 50 (for example, they had an 18% lower risk of affective and anxiety disorders). In this way, they confirmed, on a large sample of 9603 participants, the earlier findings of Jang et al. [41], who concluded that involvement in youth movements (including Scouting) has an indirect positive relationship with subjective well-being in adulthood. The effect of these good health indicators achieved by scouts is a huge public health savings [39]. Only limited research reports a negative effect of Scouting, mainly that of volunteer fatigue [42].

Our results are moderately (due to a somewhat weak moderating effect in SSA and negative verification of some of the hypotheses) consistent with previous research showing that non-formal education, and Scouting in particular, has a positive impact on children and young people’s personal and social skills [39].

Previous research has shown that young adults participating in additional sports activities or belonging to various organizations are more likely to choose task-focused coping strategies [43]. Also, in our research, scouts were characterized by a higher tendency to use a task-focused strategy and a lower tendency of avoidance compared to non-scouts. In our research, membership in the Scout Movement caused a more pronounced blocking effect of the sense of self-efficacy on the level of emotional (and therefore ineffective) stress coping style compared to non-scouts. This can be related to self-belief, self-confidence, and confidence in one’s competence. The literature indicates a positive effect on the development of psychological parameters within this group of traits and skills, mainly self-esteem, as well as social and emotional competence, for example, and the ability to solve problems effectively and to cope with difficult situations [1,2,4,5,44,45,46,47,48,49,50,51,52,53,54,55].

Why do we treat emotional control/blocking in stressful situations as part of health and adaptation? Most conceptualizations distinguish between coping strategies geared towards approaching and confronting a problem, and strategies geared towards reducing stress by avoiding direct coping. Emotion-focused coping is defined as the individual’s efforts aimed at reducing stress through emotional reactions, including emotional expression, blaming others, blaming oneself, holding back emotions and passive resignation: ‘Preoccupied with aches and pains’, ‘Tell myself that is not really happening to me’, ‘Blame myself for not knowing [what] to do’, or ‘Worry about what I am going to do’ [56]. Researchers emphasize that the emotional coping style focuses on mitigating the challenge by minimizing the emotional outcome, rather than eliminating the stressor. As a result, the challenge/problem still exists because the focus is on controlling and reducing adverse emotional effects rather than solving the problem. Therefore, the stressor remains present, causing a negative burden [57]. This leads to the conclusion that most features of the emotional coping style are mainly ineffective mechanisms, leading to dysfunctional outcomes, such as negative affect, depression, and anxiety [58,59]. Emotion-focused styles have been linked to a predisposition to increase and maintain emotional arousal in response to emotional events [60,61], or even anxiety and depression [62,63,64,65]. It appears that the ability to effectively control/regulate emotions (through, for example, a sense of self-efficacy (‘I know I can/I can do it’) may have a positive impact on mental health and minimize the risk of adaptive problems. Individuals who use active coping strategies tend to see themselves as persons in control of the situation, have a positive self-image, and take a proactive, optimistic, and self-directed approach to dealing with life stressors. On the other hand, individuals who rely on emotion-focused coping strategies, including self-blame, avoidance, and even drug or alcohol use, cope worse than individuals who adopt active strategies, such as seeking social support [66]. Avoidance-focused coping styles, such as denial and wishful thinking, on the other hand, are generally associated with greater psychological distress [67]. Thus, it should be considered that differences in the intensity of self-efficacy affect how one functions in situations of increased levels of perceived stress. Empirical evidence suggests that the ability to effectively cope with stress and emotions has important consequences for health and adaptive functioning and even resilience [68,69]. In the literature, it can also be linked to emotional regulation. Numerous studies indicate that emotional regulation is related to mental health, mainly reducing stress, depression, and personality disorders [70,71,72,73,74,75]. In adolescents, emotional dysregulation is also associated with psychological inflexibility [71]. According to the process model of emotion regulation, emotions can be regulated at five points in the emotion generation process: (a) situation selection, (b) situation modification, (c) attentional deployment, (d) cognition change, and (e) response modulation [76]. In our research, it was shown to be consistent with understanding it as a modulation of reactions directly related to goals or tasks, or as a way of inhibiting emotional states, i.e., goal-focused emotion regulation [77].

## 5. Conclusions

The ability to regulate emotions affects stress and mental health at all ages. We conclusively broadened our understanding of the role of Scouting as a moderator in coping with stress and in mental health. The studies provided detailed knowledge regarding specific mechanisms through which Scouting can influence emotional regulation and, therefore, mental health. Bandura’s self-efficacy theory suggests that self-confidence in a person’s abilities may determine their motivations and decisions; it is worth building this self-confidence, for example, in the form of self-efficacy [78].

Scouting is intended to be a method of transmitting important core values that are useful for success in a rapidly changing society. Specifically, belonging to the Scout Movement intensifies the blocking effect of self-esteem on emotions in stressful situations. Relationships between variables are evident and, although not very strong, are statistically significant. This regularity seems to be directly related to the social learning theory, which states that a person only learns new behaviors when actively involved in an activity. This is a phenomenon used in various groups supporting healthy behavior [6,7] and is also an inherent part of the concept of Scouting.

Practical implications. It seems that people join the Scout Movement to improve (perhaps seen as insufficient) personal competence. Perhaps they expect Scouting to help them control these emotions. Scouts are not always ‘different in advantage’. This is evidenced not so much by the identified moderating relationship, but rather by the fact that scouts and non-scouts did not differ in the intensity of their emotion-focused stress coping style. In the educational activities planned through participation in Scouting, the sense of self-efficacy of the participants should be assessed first, in order to anticipate the risk of using emotions to cope with stress. In the case of scouts, working on a sense of self-efficacy also more clearly reduces the risk of unconstructive emotions appearing in this type of situation than in unstructured situations. It seems that this type of relationship is realized mainly through action and, typically for Scouting, experiencing success in a group arranged educational situation.

It is important to contextualize the relatively weak moderating effect of Scouting critically. Is this effect significant in real-world applications, or does it simply reflect statistical significance? This study offers new insights that can serve as a theoretical foundation for stress management applications aimed at helping individuals utilize effective coping strategies. It suggests pathways for further research, as well as considerations for the essence of educational programs within the Scout Movement, particularly regarding elements like voluntariness, individuality, and goal awareness. These aspects may enhance the role of self-efficacy, which has emerged as a primary regulatory mechanism. For educators and staff, this means setting realistic goals based on an accurate understanding of students’ abilities, fostering supportive beliefs, and emphasizing the development of independence. This aligns with Bandura’s interpretation of self-efficacy, which highlights the importance of believing in one’s ability to organize and execute the necessary actions to manage future situations, even in the face of obstacles. Essentially, it involves a deep-seated belief in one’s capability to succeed in specific circumstances, and an assessment of one’s ability to mobilize and apply the necessary resources and skills to meet challenges. Engagement in a scout organization can thus be viewed as a fourth source of self-efficacy identified by Bandura [79]—it provides positive experiences linked to the achievement of specific goals, accompanied by emotional arousal and positive emotions like joy, satisfaction, and happiness. These experiences bolster a belief in one’s control over situations and behavior, as well as the potential for effective action in various domains [80]. In summary, Bandura’s self-efficacy theory suggests that a person’s confidence in their abilities can influence their motivations and decisions, with the social environment (such as a scout troop) offering opportunities to foster this confidence through experiences of acceptance, success, and collaboration. It also connects to the sense of agency and self-regulation, wherein individuals self-motivate and regulate their actions to achieve goals. An illustrative strategy that recognizes the significance of emotional control and self-regulation is the ‘Knowing My Emotions Program: Mental Wellness Patch Program for Juniors’ (https://www.girlscouts.org/content/dam/gsusa/forms-and-documents/activity-zone/all-ages-levels/23_GSUSA_MentalWellness_Knowing-My-Emotions-Facilitator-Guide.pdf, accessed 5 December 2024). Such initiatives are particularly worthy of recommendation, especially in light of the findings obtained.

Limitation and future research direction. The research is characterized by several limitations that may have influenced the final results. The first limitation was the difference in the size of the male and female groups involved in this study. The sample size of 683 participants is satisfactorily large, but the gender imbalance (71% female) raises concerns about generalizability. Research has shown that women tend to score higher than men on emotion-focused and avoidance-focused coping scales [81]. Additionally, the literature indicates that the moderating effects of self-esteem processes differ between girls/women and men, particularly concerning self-soothing mechanisms [82]. It is also noted that men often perceive themselves as more emotionally competent than women, which might contribute to their reluctance to engage in research studies, whereas women exhibit a greater focus on emotionality [83]. These gender differences in emotional processing and coping strategies may have influenced the results obtained in various studies. It is crucial to consider these factors and draw conclusions with caution, recognizing how gender dynamics can affect both self-perception and participation in research.

Conducting the research online proved to be another limitation. The online data collection method is practical but introduces potential biases and limits control over the research process. A paper-and-pencil study would have offered a greater control of the research procedure. Now, we also consider the lack of information on education to be a limitation (although the age of the respondents was unlikely to lead to significant differences in this area)—according to the assumptions of the Polish educational system, individuals between the ages of 16 and 21 are typically classified as high school students or first-year university students. However, it is important to monitor this data in the future to assess the potential impact of the school type and its environment on various outcomes. By controlling for these factors, researchers can gain a clearer understanding of how different educational settings influence students’ experiences and development during this critical age range.

It should also be borne in mind that, in this paper, we use the concept of an emotion-based stress coping strategy as an ineffective strategy, whereas other approaches treat other strategies, such as seeking emotional social support, positive reinterpretation, acceptance, denial, or turning to religion [84], as emotional strategies.

It should also be remembered that research using volunteers requires caution when generalizing the results, because there may be significant differences between people who decided to take part in the study and those who did not [85]. Another limitation of this study is the geographical context in which it was conducted. To verify the results, they need to be repeated in other contexts.

In social science research, “moderators” are variables that can influence the direction and strength of the relationship between two or more variables. To establish external validity, it is essential that the relationships identified in a study hold true across different settings and populations. Research often examines the relationships between variables to understand how one variable impacts another. However, interaction between these variables may be influenced by various factors or “moderators”. These can include specific characteristics of the participants or contextual elements of the study, which may alter the correlational relationship. As a result, moderators indicate that the relationship between two variables might not generalize across all subgroups or conditions, thereby affecting external validity. For instance, if a study group has a significant predominance of women, this demographic makeup could influence the moderation effects observed. Therefore, future studies should aim to include a more balanced representation of participants, such as increasing the number of men studied, to achieve more comprehensive results.

It seems that, for future studies, it would be useful to specify the length of involvement in the organization (seniority in the organization), the level of involvement, and to detail the nature of the activities of the respondents affiliated with the Scouting organization. It also seems worthwhile to include in future analyses the analytical strategy of combining data and modelling growth curves. Moderating effects can be used to examine demographic predictors, such as the intersection (combination) of race/origin and gender effects, or psychological constructs, such as protective factors for different outcomes. Longitudinal research is also suggested to examine the long-term impact of Scouting on mental health, as well as examining the role of additional variables, such as cultural differences, socioeconomic status, or level of involvement in Scouting activities, to better understand their impact. It is also necessary to compare other models of non-formal education to compare their effectiveness with Scouting.

In summary, this study represents a unique moderation analysis of a system of variables that has not been previously explored in the existing literature. It highlights the significance of self-efficacy concerning emotional regulation and draws attention to coupling mechanisms that have yet to receive much empirical focus. The findings suggest that these mechanisms can offer developmental benefits to members of the Scout Movement, and we hope they may also apply to formal education, particularly in enhancing adaptation to stressful situations through effective emotion regulation. Furthermore, this study indicates that informal education, with Scouting as a leading example, is an ideal context for implementing these strategies [86]. These conclusions are vital for developing public policies that encourage greater involvement of local communities in non-formal education initiatives, fostering creativity, critical thinking, and adaptability skills [87,88,89,90], that are essential in our rapidly changing world.

## Figures and Tables

**Table 1:** Descriptive statistics and results of the Kolmogorov–Smirnov test for the whole sample and compared subgroups.

Variable	*M*	*SD*	*Sk*	*Kurt*	*Z*
Whole sample (*N* = 683)					
Age	17.61	1.62	0.79	−0.51	0.21 ***
Sense of Self-Efficacy	29.38	4.85	−0.23	−0.10	0.08 ***
Self-esteem	26.23	5.81	−0.08	−0.55	0.06 ***
Task-focused coping style	55.42	9.45	−0.17	−0.08	0.05 **
Emotion-focused coping style	49.79	12.20	−0.09	−0.47	0.03
Avoidance-focused coping style	47.90	10.76	−0.05	−0.41	0.05 **
Members of the Scout Movement (*n*_1_ = 324)					
Age	17.82	1.67	0.62	−0.82	0.21 ***
Sense of Self-Efficacy	29.81	4.68	−0.33	0.31	0.08 ***
Self-esteem	26.19	5.67	−0.09	−0.68	0.08 ***
Task-focused coping style	56.58	9.31	−0.23	0.07	0.06 *
Emotion-focused coping style	49.61	11.97	−0.21	−0.47	0.06 *
Avoidance-focused coping style	46.99	10.65	0.08	−0.36	0.04
Non-members of the Scout Movement (*n*_2_ = 359)					
Age	17.42	1.55	0.96	−0.09	0.21 ***
Sense of Self-Efficacy	28.99	4.98	−0.14	−0.36	0.08 ***
Self-esteem	26.27	5.95	−0.08	−0.45	0.05 *
Task-focused coping style	54.36	9.46	−0.12	−0.15	0.05 *
Emotion-focused coping style	49.96	12.42	0.01	−0.48	0.05
Avoidance-focused coping style	48.72	10.81	−0.18	−0.38	0.06 *

*** *p* < 0.001; ** *p* < 0.01; * *p* < 0.05.

**Table 2:** Student’s *t*-test results for independent samples.

Variable	Group	*M*	*SD*	*F*	*t*	95%CI	
						LB	UB
Sense of Self-Efficacy	Scouts	29.81	4.68	2.43	2.21 *	0.92	1.55
	Non-scouts	28.99	4.98				
Self-Esteem	Scouts	26.19	5.67	0.27	0.17	−0.95	0.80
	Non-scouts	26.27	5.95				
Task-focused Coping Style	Scouts	56.58	9.31	0.15	3.09 **	0.81	3.63
	Non-scouts	54.36	9.46				
Emotion-focused Coping Style	Scouts	49.61	11.97	0.25	0.37	−2.19	1.49
	Non-scouts	49.96	12.42				
Avoidance-focused Coping Style	Scouts	46.99	10.65	0.02	2.10 *	−3.34	−0.11
	Non-scouts	48.72	10.81				

** *p* < 0.01; * *p* < 0.05

**Table 3:** Results of the *r*-Pearson correlation (*N* = 683).

	Sense of Self-Efficacy	Self-Esteem
Self-Esteem	0.51 ***	-
Task-focused Coping Style	0.60 ***	0.41 ***
Emotion-focused Coping Style	−0.38 ***	−0.59 ***
Avoidance-focused Coping Style	0.10 *	0.06

*** *p* < 0.001; * *p* < 0.05

**Table 4:** Results of moderation analyses.

Model	X	Y	*F*	*R* ^2^	∆*R* ^2^	*B*_1_	*B*_2_	*Int*	95%CI	
									LB	UB
1	X1	Y1	130.44 ***	0.37	0.001	1.22 ***	1.11 ***	−0.11	−0.35	0.12
2	X1	Y2	5.25 **	0.02	0.005	0.05	0.37 **	0.33	−0.01	0.66
3	X1	Y3	41.21 ***	0.15	0.008	−1.21 ***	0.77 ***	0.44 *	0.09	0.80
4	X2	Y1	51.73 ***	0.19	0.004	0.78 ***	0.57 ***	−0.21	−0.43	0.01
5	X2	Y2	2.63 *	0.01	0.001	0.05	0.17	0.12	−0.16	0.40
6	X2	Y3	120.44 ***	0.35	0.002	−1.12 ***	−1.33 ***	−0.21	−0.47	0.05
7	X2	X1	82.39 ***	0.27	0.001	0.42 ***	0.43 ***	0.01	−0.10	0.11

*** *p* < 0.001; ** *p* < 0.01; * *p* < 0.05. Annotation. ∆*R*^2^—change in coefficient *R*^2^; *B*_1_—non-standardized regression value between variables for the group of members of the Scout Movement; *B*_2_—non-standardized regression value between variables for the group of non-members of the Scout Movement; *Int*—interaction effect value; LB—lower confidence interval; UB—upper confidence interval. Variables: X1—Sense of Self-Efficacy; X2—Self-esteem; Y1—Task-focused coping style; Y2—Avoidance-focused coping style; Y3—Emotion-focused coping style.

**Table 5:** Hypotheses verification—resume.

Hypotheses	Result of Verification	Indicator
H1	Confirmed	*t*(683) = 2.21; *p* < 0.05
H2	Rejected	*t*(683) = 0.17; *p* > 0.05
H3.1	Confirmed	*t*(683) = 3.09; *p* < 0.01
H3.2	Rejected	*t*(683) = 0.37; *p* > 0.05
H3.3	Confirmed	*t*(683) = 2.10; *p* < 0.05
H4.1	Rejected	*Int* = −0.11; *p* > 0.05
H4.2	Confirmed	*Int* = 0.44; *p* < 0.05
H4.3	Rejected	*Int* = 0.33; *p* > 0.05
H5.1	Rejected	*Int* = −0.21; *p* > 0.05
H5.2	Rejected	*Int* = 0.12; *p* > 0.05
H5.3	Rejected	*Int* = −0.21; *p* > 0.05
H6	Rejected	*Int* = 0.01; *p* > 0.05

## Data Availability

The data presented in this study are available on reasonable request from the corresponding author.
